# Observations on an Indigenous Remedy for Cancer, with a Copy of the Prescription

**Published:** 1814-08

**Authors:** Leonard Gillespie

**Affiliations:** Physician in the Royal Navy. Bath


*For the Medical and Physical Journal.*

### *Observations on an indigenous Remedy for Cancer, with a Copy of the Prescription; by* LEONARD GILLESPIE, M.D.

THAT savage nations are possessed of secrets, as to the nature of plants and their virtues, powerful in destroying and in preserving life, the testimony of many enlightened

---

travellers sufficiently demonstrate: hence the study and investigation of indigenous medicine and surgery, amongst the savage natives of tropical climates, abounding as these countries do with vegetable simples of the most efficacious nature in many diseases, has always appeared to me very interesting, and in my practice for many years in the West-India Islands, has proved of the utmost benefit to me.

These remarks are premised to the contents of the annexed paper, containing an indigenous African cure for cancer, communicated to me by Mr. Henry Haffey, late a Member of Council, and Colonel of Militia in the Island of St. Vincent, of which he was one of the oldest planters. Mrs. Haffey, who is well acquainted with the Carribean plants, joins her husband in asserting that she was an eye-witness of the two cures performed, mentioned in this paper: whether or not these sores were true cancers, it would be difficult to ascertain, but the treatment of them as such by the medical men consulted, together with the destruction of the nose of the patient in both instances, are sufficient proofs of the malignant nature of those ulcerations.

The plants here mentioned are very common in the West-India Islands: the physic-nut is a shrub abounding with a tenacious juice; the leaves are used in infusion internally as a purgative and febrifuge, and are used externally in decoction as a wash for ulcers. The experienced Dr. Granger, in his Essay on West-India Diseases, in treating of ulcers of the legs (p. 86), says, "After washing the ulcers with vitriol water, French physic-nut leaves should be pounded and applied fresh to the sore. I have known that application succeed, when the most pompous prescriptions of the shops have failed."

The other plant, the thistle of the Islands, is totally different from the thistle of Europe: it is a succulent plant very common in the Islands.

As a remedy for cancer, Goclioke*, in a dissertation, recommended the juice of the tomentous thistle of Europe; and it is an article in the materia medica of the Russian Pharmacopœia.

Mr. Haffey has, at my solicitation, sent to St. Vincents for some of this preparation, and when it arrives, he will take care to send some of it for trial to a surgeon versed in the treatment of cancers.

*Bath,* LEONARD GILLESPIE,

*June* 21, 1814. *Physician in the Royal Navy.*

P.S. I have given the Linnæan and French names of the plants.

---

* De Onopordo, Carcinomatis Avorrunco, 1739.

*A Cure*

*A Cure for Cancers.*

Half a pint of the milk of physic-nut,*
Half a pint of the milk of thistle,†
1 oz. of Castile soap, cut fine,
A wine glass of rum.

The above to be well mixed and dried in the sun until it turns to the consistence of salve, to be spread on lint and applied to the part affected, first bathing it with a decoction of the leaves of the physic-nut; those of the English plantain are usually substituted.

In the year 1794, a cancer appeared in the nose of a young female, servant of mine, who was treated by medical men for twelve months and more, but the complaint increased, and her nose was lost. She found out an old African woman, who undertook to cure her, which she effected by the above recipe. She is now living, and in good health.

In the year 1796, a cancer appeared on the nose of a young male servant belonging to me. He also lost his nose, but in other respects is in good health, the progress of the cancer being stopped by the above recipe.

The patient should live low, not eat much animal food and no fish. H. H.

* Tutropa multifida, Linn. Medicinier of the French.

† Argemone Mexicana, Linn. Yellow thistle, the seed of which is in common use in the West-India Islands as an excellent yet mild emetic and purgative in dysentery, remittent and intermittent fevers.

